# Supplementary material for: Effect of Stepwise Exposure to High-Level Erythromycin on Anaerobic Digestion
Source: Molecules. 2024 Jul 25;29(15):3489. doi: 10.3390/molecules29153489 (PMC11313690; doi:10.3390/molecules29153489)
Supplement: Supplementary file 1 [file molecules-29-03489-s001.zip › molecules-3068197-supplementary.pdf]

Effect of stepwise exposure to high-level erythromycin on anaerobic digestion

Yanxiang Zhang [1;] Chunxing Li [2], Xinyu Zhu [3]* and Irini Angelidaki [4]

[1] *School of Environmental and Material Engineering, Yantai University, Yantai, Shandong, 264005, China*

[2] *State Key Laboratory of Pollution Control and Resource Reuse, School of the Environment, Nanjing University, Nanjing 210023, China*

[3] *Key Laboratory of Coastal Environment and Resources of Zhejiang Province, School of Engineering, Westlake University, Hangzhou 310030, China*

[4] *Department of Chemical and Biochemical Engineering, Technical University of Denmark, Kgs. Lyngby DK-2800, Denmark*

* *Correspondence: zhuxinyu@westlake.edu.cn*

Table S1. qPCR primer sequences for antibiotic resistance genes analysis.

Resistance mechanism	gene	5' to 3'	Reference
esterase gene	*ereA*	F: CCTGTGGTACGGAGAATTCATGT<br>R: ACCGCATTCGCTTTGCTT	[1-3]
	ereB	F: GCTTTATTTCAGGAGGCGGAAT<br>R: TTTTAAATGCCACAGCACAGAATC	
phosphorylase gene	*mphA-01*	F: CTGACGCGCTCCGTGTT<br>R: GGTGGTGCATGGCGATCT	
	mphB	F: CGCAGCGCTTGATCTTGTAG<br>R: TTACTGCATCCATACGCTGCTT	
methylase gene	*ermA*	F: TTGAGAAGGGATTTGCGAAAAG<br>R: ATATCCATCTCCACCATTAATAGTAAACC	
	ermB	F: TAAAGGGCATTTAACGACGAAA<br>R: TTTATACCTCTGTTTGTTAGGGAATTGAA	
	ermC	F: TTTGAAATCGGCTCAGGAAAA<br>R: ATGGTCTATTTCAATGGCAGTTACG	
	ermX	F: GCTCAGTGGTCCCCATGGT<br>R: ATCCCCCCGTCAACGTTT	
efflux pump gene	*mefA*	F: CCGTAGCATTGGAACAGCTTTT<br>R: AAACGGAGTATAAGAGTGCTGCAA	
	mefE	F: CGTATTGGGTGCTGTGATTG<br>R: TATGCACAGGCGTTCCATTA	

References

[1] M.T. Guo, Q.B. Yuan, J. Yang, Ultraviolet reduction of erythromycin and tetracycline resistant heterotrophic bacteria and their resistance genes in municipal wastewater, Chemosphere, 93 (2013) 2864-2868.

[2] M. Liu, R. Ding, Y. Zhang, Y. Gao, Z. Tian, T. Zhang, M. Yang, Abundance and distribution of Macrolide-Lincosamide-Streptogramin resistance genes in an anaerobic-aerobic system treating spiramycin production wastewater, Water Res, 63 (2014) 33-41.

[3] Y. Chen, P. Li, Y. Huang, K. Yu, H. Chen, K. Cui, Q. Huang, J. Zhang, K. Yew-Hoong Gin, Y. He, Environmental media exert a bottleneck in driving the dynamics of antibiotic resistance genes in modern aquatic environment, Water Res, 162 (2019) 127-138.
